# Mapping Conservation Biological Control and IPM Research (2000–2025): A Bibliometric Analysis of Natural Enemies and Habitat Management

**DOI:** 10.3390/insects17050447

**Published:** 2026-04-23

**Authors:** Moazam Hyder, Farman Ullah, Abdul Basit, Inzamam Ul Haq, Tijjani Mustapha, Zaib Un Nisa, Xiangyun Cai, Huiping Liu, Youming Hou

**Affiliations:** 1 State Key Laboratory of Agricultural and Forestry Biosecurity, Key Laboratory of Biopesticides and Chemical Biology, MOE, College of Plant Protection, Fujian Agriculture and Forestry University, Fuzhou 350002, China; 2 State Key Laboratory for Biology of Plant Diseases and Insect Pests, Key Laboratory of Natural Enemy Insects, Ministry of Agriculture and Rural Affairs, Institute of Plant Protection, Chinese Academy of Agricultural Sciences, Beijing 100193, China; 3 Xianghu Laboratory, Institute of Bio-Interaction, Hangzhou 311258, China; farmanullah787@gmail.com; 4 Cotton Research Institute, Multan 60000, Pakistan

**Keywords:** biological control, pest management, sustainability, natural enemies, bibliometric analysis

## Abstract

Concerns regarding the safety, environmental impacts, and long-term sustainability of pesticide-dependent crop protection have intensified interest in biological control, which suppresses pest populations using natural enemies (predators, parasitoids, and pathogens) within integrated pest management (IPM) programs. This bibliometric study maps the development of biological control research from 2000 to 2025 using records retrieved from the Web of Science Core Collection. The publication trends, collaboration structures, leading countries and institutions, core journals, keyword co-occurrence and clustering, citation bursts, and influential cited references were examined using CiteSpace and VOSviewer. The results show a pronounced increase in publication output after 2011, indicating rapid expansion and consolidation of the field in the last decade. Keyword analyses reveal a thematic shift toward ecosystem-based framing, reflected by the growing prominence of terms such as ecosystem services, habitat management, and ecological intensification, which emphasize landscape- and management-oriented approaches to enhancing pest suppression. Cited-reference patterns highlight the persistent influence of the foundational literature on habitat manipulation, landscape complexity, and conservation biological control. Despite the field’s growth, research gaps remain in integrating biological control with emerging bioengineering tools and explicitly accounting for climate-driven variability across regions and production systems.

## Simple Summary

This review focuses specifically on conservation biological control, which emphasizes utilizing habitat management and landscape complexity to enhance natural-enemy populations, rather than classical or augmentative biological control. This study reviews global research activity on biological control from 2000 to 2025 using a bibliometric approach based on the Web of Science Core Collection. With CiteSpace and VOSviewer, we mapped who is publishing, where the work is concentrated, which journals and references shape the field, and how key topics are changing over time. Research output increased sharply after 2011. The dominant themes increasingly emphasize how farming landscapes are managed, for example through habitat design that supports natural enemies rather than focusing only on individual control agents. Highly influential studies continue to guide this direction, especially work on habitat manipulation and landscape complexity. Important opportunities remain to connect biological control more directly with climate-resilient planning and emerging biotechnological tools.

## 1. Introduction

Agriculture constitutes the cornerstone of global food provision; however, it faces daunting challenges due to pest invasions damaging 20–40% of crops every year globally, further compromising economic sustainability and food accessibility in at-risk communities [1,2]. The dependence on synthetic chemical pesticides, which is based on historical use, has generated short-term gains in protecting crop yields but this comes at a high price in the long run, including drawbacks such as increased pesticide resistance of pest species [3,4,5], contamination of soil and water resources [6,7,8], as well as negative effects to human health and non-target organisms such as pollinators and beneficial insects [9,10,11]. Such consequences have prompted a clumsy paradigm change that needs tightening to minimize ecological footprints and also support productivity [12,13].

Biological control is broadly categorized into three approaches: classical (introduction of exotic natural enemies), augmentative (mass release of natural enemies), and conservation (habitat manipulation to protect and enhance existing natural enemies). The present bibliometric analysis focuses primarily on conservation biological control, as the retrieved literature predominantly addresses habitat management, landscape complexity, and ecosystem services [14,15,16,17]. Using biological control is also a crucial part of integrated pest management (IPM), as it employs the use of natural enemies along with other sustainable practices to minimize the use of pesticides while saving the beneficial species. Recent studies have shown that the use of ladybird beetles and lacewings as biological control agents in the control of aphids and other pests has been very successful [18,19]. Predators such as ladybirds and lacewings, along with mirid bugs, serve an important function of improving crop yield and quality by controlling aphids and whiteflies [20,21]. Tangible success stories illustrate the effectiveness of biological control across diverse systems. For example, classical biological control programs have successfully managed lepidopteran pests such as *Cactoblastis cactorum* in extensive crops using host-specific parasitoids [22]. In intensive fruit and vegetable production, augmentative releases of *Trichogramma* spp. have been widely used for the control of lepidopteran pests [23]. Additionally, entomopathogenic fungi such as *Beauveria bassiana* and *Metarhizium anisopliae* are increasingly used to suppress a wide range of arthropod pests, offering viable alternatives to chemical insecticides [24]. While these approaches are well-documented, the present bibliometric dataset indicates that conservation biological control focusing on habitat manipulation and landscape management dominates the recent literature. Research also shows that the provision of adequate habitat and diverse flowering plants in the agroecosystem improves the biological control function of natural enemies [25,26,27,28,29].

The control of pests can depend on habitat space, prey availability, and other factors [30,31]. Enhancing the habitat of natural enemies is called conservation biological control, and due to the planting of flower strips, habitat preservation, and the possibility of increasing biological conservation control, this method has been receiving more attention [20,32,33,34]. Protecting the flowering resources of the predators and parasitoids is the best way to increase their pest control [25,35]. Climate change has the potential to alter the above relationships by disrupting the balance between pests and their natural enemies [2,16]. Generalist predators, such as ladybirds that can adjust to variable prey availability in response to climate change, are crucial for maintaining biological control efficacy and therefore maintenance of their populations by decreasing pesticide use and increasing non-crop habitats [36,37,38]. Increasing ladybird biocontrol through higher edge density in alfalfa fields enhances population levels of ladybirds and thereby the level of biocontrol they provide; thus, their conservation becomes a strategy for mitigation against habitat fragmentation [39,40]. Innovative pest control strategies that blend the use of natural enemies and the changing ecology of the pests are essential to conserve the effectiveness of biological control.

The ongoing transformation of global agriculture signifies the importance of biological control in contemporary farming systems. The use of environmentally safe alternatives to synthetic pesticides has been driven by the need to protect the environment and legal restrictions. In addition to pest relief, biological control has multiple advantages, such as preserving biodiversity, improving soil quality, and building the sustainability of farming systems [12,18]. Furthermore, biological control positively fosters agroecosystems, addressing food security and environmental sustainability [41,42].

Bibliometric analyses have been crucial in documenting important trends, prominent authors, and research deserts in the field of biological control. The increased volume of literature, particularly after 2011, signifies a global transition towards the sustainability of pest control practices. Research highlights the need for the incorporation of natural enemies into integrated pest management (IPM) systems, with an increased focus on biopesticides, biofungicides, and plant growth-promoting rhizobacteria (PGPR) to control phytopathogens [15,43]. It is evident that the field of pest control is moving away from the use of synthetic pesticides towards a more holistic, environmentally friendly approach.

The successful application of biological control methods within integrated pest management continues to encounter challenges. These include the variation in natural enemies across different regions, as well as the need for improved mass-rearing techniques [44,45]. This study is intended to be a bibliometric analysis of biological control for the years 2000 to 2025, noting trends, prominent authors, and major references. It is important that the use of biological control be coupled with other methods of IPM for the development of agriculture to be sustainable and to address the challenges of biodiversity loss and food insecurity [46,47].

## 2. Materials and Methods

### 2.1. Data Collection

Bibliometric information on the research was obtained from the Web of Science (WoS) Core Collection database; this tool was chosen due to its extensive coverage of high-quality, peer-reviewed materials in the agricultural and ecological fields. The query was TS = (“biological control” OR biocontrol OR natural enemies OR predators OR parasitoids OR conservation) AND TS = (integrated pest management OR IPM OR sustainable agriculture OR pest control), and the search was conducted on 15 December 2025. The time frame was restricted from the beginning of the year 2000 to the end of the year 2025 to reflect the past 20 years of research trends and the proposed recent forecasts. English-language articles, reviews and proceedings papers were considered, but not books, editorials or letters. This gave a dataset of 1212 records that were downloaded in full-record format with cited references that could be further analyzed. Data cleaning facilitated the elimination of characteristics such as duplication and irrelevant records using WoS tools and hand-checking as far as relevancy in the context of biological control and natural enemies in agricultural settings is concerned.

### 2.2. Bibliometric Analysis Software

CiteSpace (version 6.2.R6), a Java-based application designed for visualizing and analyzing patterns and trends in scientific literature, was employed to conduct the bibliometric analysis. The software was selected for its advanced capabilities in generating co-authorship networks, co-citation networks, keyword co-occurrence maps, and citation-burst detection.

The following parameters were applied: the time span covered 2000 to 2025 with one-year intervals; the node types included authors, institutions, countries, keywords, and references; node selection was based on the g-index (k = 25) with the top N% per slice; and network pruning was performed using the Pathfinder algorithm together with pruning of sliced networks to simplify the visualizations.

Keyword clustering and co-occurrence networks were used to cross-validate the results. The data were further processed with VOSviewer (version 1.6.20), where the minimum occurrence threshold for keywords was set to 5 and the overlay visualization mode was applied.

### 2.3. Analysis Indicators

Several bibliometric measures were used to estimate the research landscape: Publication output: Annual publication numbers were computed and graphed to determine the growth patterns, and forecasts made in 2025 using linear regression through data between 2020 and 2025. Authors and co-authors: Co-authorship networks were created to determine central authors based on betweenness centrality (the threshold was set to 0.01) and the number of publications. Countries and institutions: Both the country’s collaboration networks and institutional co-authorship networks were evaluated based on centrality scores and total publications. Journals: Co-citation networks were generated to exemplify the most important journals, and the impact factors were obtained via Journal citation reports 2024–2025. Keywords: Keyword co-occurrence, clustering, and citation-burst analyses were performed, and the most popular keywords were ranked by frequency and centrality; clusters were named with the help of LLR algorithm. Cited references: Co-citation networks and top-cited references were examined in terms of citation counts and centrality to find foundational works. All the visualizations were to be exported out of CiteSpace and then enhanced in Adobe illustrator to make them clear. R (version 4.5.2) was used to carry out statistical analyses such as trend regressions to aid in interpretations. Such an approach guarantees the soundness of the analysis of the biological control research sphere with the ability to reproduce.

## 3. Results

### 3.1. Publication Output Analysis

**Figure 1:**
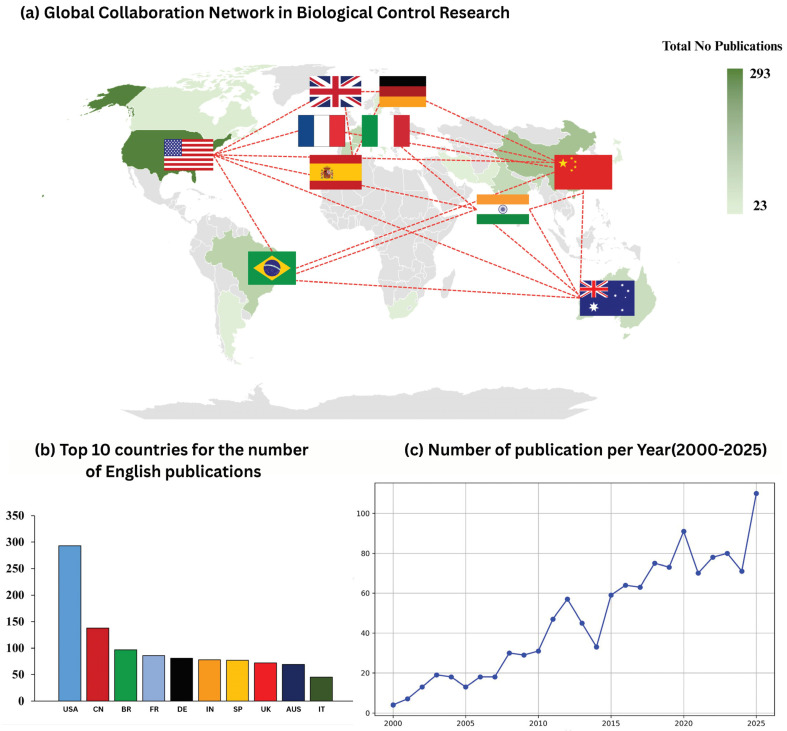
Global publication landscape of biological control/IPM research (2000–2025). (**a**) Global collaboration network map showing major publishing countries and their collaboration links; country shading indicates total publication volume, and connecting lines indicate cross-country co-authorship relationships. (**b**) Top 10 countries by number of English-language publications in the dataset (e.g., USA, China, Brazil, France, Germany, India, Spain, UK, Australia, Italy). (**c**) Annual publication output (2000–2025), showing a clear acceleration after ~2011 and sustained high productivity in the most recent years.

A total of 1212 Web of Science-indexed publications on biological control, natural enemies, and integrated pest management (IPM) were retrieved for the period 2000–2025. The annual publication trajectory demonstrates a clear long-term expansion of the field (Figure 1c), with three discernible phases of development. In the early period (2000–2010), publication output remained low and increased gradually, indicating that research activity was still emerging and comparatively fragmented. From 2011 onward, output increased more rapidly, marking a transition into a growth phase. This acceleration is evident in the sustained rise during 2011–2016, culminating in approximately 64 publications in 2016 (Figure 1c). After 2016, annual output remained consistently elevated, with a further intensification in the most recent period. The dataset shows a pronounced increase around 2020 (approximately 91 publications), followed by continued high productivity through 2021–2024. By 2025, annual output reached approximately 110 publications, representing the highest level observed in the series (Figure 1c). The publication dynamics indicate that biological control research has shifted from a relatively limited early literature base to a mature and rapidly expanding domain in the last decade. The sustained high output after 2015, together with the recent peak, reflects the consolidation of biological control and IPM as a major research area within agricultural and ecological sciences, with increasing attention to natural-enemy-based pest regulation at both local and landscape scales (Figure 1c).

### 3.2. Central Authors and Collaborators

**Figure 2:**
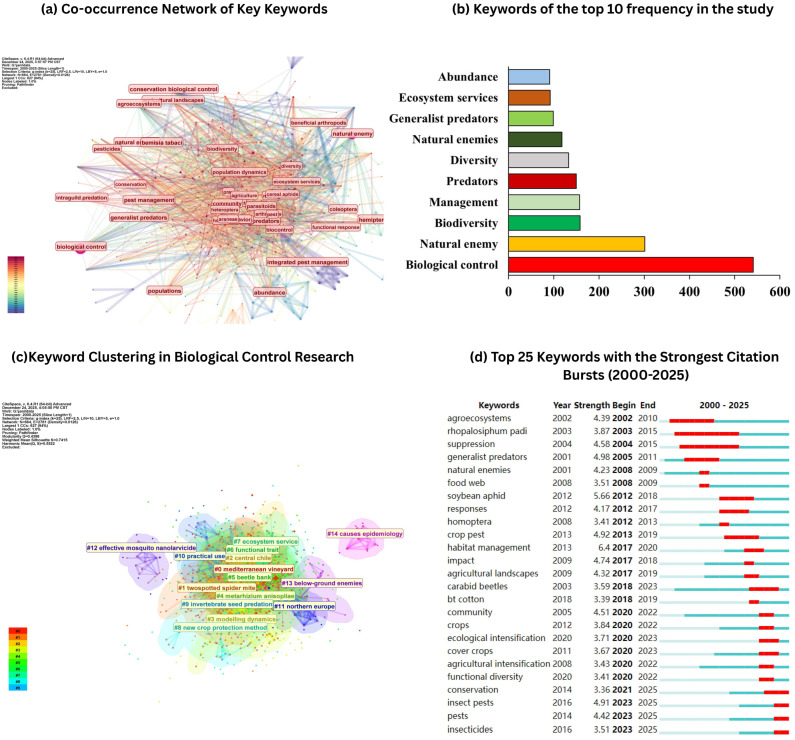
Keyword structure and emerging trends in biological control/IPM research (2000–2025). (**a**) Keyword co-occurrence network of high-frequency terms, indicating how major concepts co-appear across the literature. (**b**) Top 10 keywords by frequency in the dataset. (**c**) Keyword clustering map showing major thematic communities within the field. (**d**) Top 25 keywords with the strongest citation bursts, indicating periods of rapidly increasing attention from 2000 to 2025.

The co-authorship analysis highlights a set of prolific authors who have contributed substantially to the development of biological control and integrated pest management (IPM) research during 2000–2025 (Figure 2b; Table 1). Desneux, Nicolas ranked first in publication output (*n* = 13), followed by Birkhofer, Klaus (*n* = 7) and Tscharntke, Teja (*n* = 7). A second tier of productive contributors included Gurr, Geoff M (*n* = 6), Lu, Yanhui (*n* = 6), and Zanuncio, Jose Cola (*n* = 6). Additional leading authors with *n* = 5 publications each were Benelli, Giovanni, Landis, Douglas A, Albrecht, Matthias, and Traugott, Michael (Table 1). Collectively, these authors represent influential research capacity spanning applied pest management, agroecology, and biodiversity-based pest suppression. Network centrality values for the leading authors were low overall (0.00–0.01; Table 1), indicating that the author collaboration structure is distributed across multiple sub-communities, rather than being coordinated through a small number of strong bridging individuals. While several authors (e.g., Desneux, Birkhofer, Gurr, and Traugott) displayed non-zero centrality values (0.01), the magnitude suggests limited brokerage between distinct collaboration clusters. This pattern is consistent with a research field characterized by topic- and region-specific teams, where collaboration tends to occur within relatively cohesive groups and expands outward through a broader network of parallel clusters rather than through a single dominant hub (Figure 2b). The visualization of the co-authorship network further supports this interpretation, showing dense local connections within author groups and comparatively fewer high-centrality connectors spanning the entire network (Figure 2b). Such a structure is typical of mature interdisciplinary domains in which multiple thematic streams such as conservation biological control, landscape complexity, pest enemy dynamics, and applied crop pest case studies develop in parallel, with periodic cross-linking through shared concepts, methods, and synthesis studies. The results indicate that leadership in biological control/IPM research is concentrated among a set of productive authors, but intellectual and collaborative influence is distributed across several interconnected clusters rather than dominated by a centralized author core (Figure 2b; Table 1).

### 3.3. Analysis of Research Countries

Country-level bibliometric analysis demonstrates that biological control and integrated pest management (IPM) research is strongly international in both productivity and collaboration (Figure 1a,b). Across the 2000–2025 dataset, the United States was the leading contributor with 293 publications, followed by China (138) and Brazil (97) (Figure 1b). A second group of high-output countries included France (86), Germany (81), India (78), and Spain and the United Kingdom with comparably strong publication volumes (Figure 1b). Australia and Italy also ranked among the top contributors, indicating sustained research engagement across North America, Europe, Asia, South America, and Oceania (Figure 1b). This distribution confirms that biological control research has developed as a globally relevant field, with major publication centers spanning both long-established research economies and rapidly expanding agricultural research systems. The global collaboration network further illustrates extensive cross-national connectivity among leading contributors (Figure 1a). Collaboration links are especially evident among the principal publication hubs, indicating that the field is supported by multi-country research partnerships rather than isolated national efforts. The presence of multiple dense collaboration pathways suggests that biological control and IPM research questions—often requiring ecological generalization, multi-regional validation, and diverse crop–pest contexts—are frequently addressed through cooperative, international study designs (Figure 1a). In addition, the prominence of countries across different agroecological zones implies that research is being shaped by both temperate and tropical agricultural priorities, including pest invasion dynamics, landscape management, and the optimization of natural-enemy communities under distinct production systems.

### 3.4. Analysis of Research Institutions

Institution-level analysis indicates that biological control and integrated pest management (IPM) research is led by a concentrated set of highly productive organizations that also occupy key positions in the collaboration network (Figure 2a; Table 2). INRAE ranked first with 65 publications and a centrality of 0.14, reflecting both high output and a strong connective role within the institutional network. The United States Department of Agriculture (USDA) ranked second with 52 publications and the highest centrality value (0.17) among the top institutions, indicating a pronounced bridging function that links multiple collaboration clusters. The University of California System followed with 35 publications and a centrality of 0.11, confirming its substantial contribution and influence in shaping research directions and collaborative activity. Major institutions from Asia were also prominent. The Chinese Academy of Agricultural Sciences (CAAS) contributed 33 publications (centrality 0.06), while the Indian Council of Agricultural Research (ICAR) produced 32 publications (centrality 0.06), demonstrating strong institutional capacity in two of the largest agricultural research systems globally. European research leadership was further reflected by the Centre National de la Recherche Scientifique (CNRS) with 29 publications and a centrality of 0.08, reinforcing the role of national research organizations in sustaining high-volume, collaborative biological control programs. Additional institutions contributing notably to the field included the Universidade Federal de Viçosa (28 publications; centrality 0.01), the Institute of Plant Protection (25; 0.04), the State University System of Florida (25; 0.03), and the University of Florida (22; 0.02) (Table 2).

The institutional collaboration visualization (Figure 2a) suggests a network characterized by several large hubs connected through multiple cross-institutional links rather than a single dominant center. Centrality values support this structure, with USDA, INRAE, and the University of California System acting as principal connectors, while CAAS, ICAR, and CNRS serve as major regional anchors with moderate bridging capacity. This configuration indicates that biological control/IPM research advances through a combination of (i) high-output institutional hubs that sustain large research portfolios and (ii) networked collaborations that facilitate cross-system knowledge transfer, comparative studies, and synthesis across crop–pest contexts (Figure 2a).

### 3.5. Key Journals in Biological Control Research

The journal co-citation analysis identifies a concentrated set of core outlets that collectively constitute the intellectual knowledge base of biological control and integrated pest management (IPM) research (Figure 2c,d). The most frequently co-cited journals include *Annual Review of Entomology* and *Biological Control*, indicating that the field is shaped by both high-impact synthesis literature and specialized applied research focused on natural enemies, pest–enemy interactions, and implementation of biological control in agricultural systems (Figure 2d). The prominence of these sources suggests that conceptual development in the domain relies heavily on integrative reviews and theory-building contributions, alongside empirically grounded studies evaluating biological control agents and strategies. A second tier of highly influential journals comprises *Ecology Letters*, *Journal of Pest Science*, *Entomologia Generalis*, and *Agriculture, Ecosystems & Environment* (Figure 2d). These journals reflect the strong integration of ecological theory with pest management practice, particularly through work addressing biodiversity-mediated pest suppression, landscape complexity, habitat management, and ecosystem service delivery in agroecosystems. The presence of *Ecology Letters* indicates that biological control research engages broadly with high-level ecological debates and quantitative synthesis, whereas *Agriculture, Ecosystems & Environment* highlights the applied landscape and agroecosystem management dimension that supports conservation biological control within IPM frameworks. Additional influential journals in the co-citation network include the *Journal of Economic Entomology*, *Entomologia Experimentalis et Applicata*, *Ecology*, and *Journal of Applied Ecology* (Figure 2d). Their co-citation prominence underscores the field’s methodological diversity, spanning fundamental populations and community ecologies, experimental evaluation of pest control interventions, and economically relevant entomological research that links ecological outcomes to crop protection objectives. Together, the co-cited journal set demonstrates that biological control/IPM scholarship is anchored by a well-defined publication ecosystem that bridges foundational ecological science, rigorous synthesis, and applied entomology.

### 3.6. Analysis of Cited References

Cited-reference analysis was conducted to identify the foundational studies that have structured the intellectual base of biological control and integrated pest management (IPM) research in the 2000–2025 dataset. The co-citation network (Figure 2) and the ranking of highly cited works (Table 3) together reveal a knowledge base dominated by landscape-scale ecological theory, habitat management for conservation biological control, and quantitative syntheses assessing how agroecosystem structure shapes pest suppression and natural-enemy communities. The citation counts reported in Table 3 represent the Web of Science “Times Cited” values at the time of data extraction (15 December 2025) and therefore capture broad scholarly influence while also reflecting the time advantage of older publications.

Among the most influential contributions, Douglas A. Landis [48] ranks first (“Habitat management to conserve natural enemies of arthropod pests in agriculture”), reflecting the centrality of habitat manipulation as a mechanism to conserve and enhance natural enemies in cropping systems (Table 3). A second major pillar of the literature emphasizes landscape-scale processes and enemy diversity, exemplified by Teja Tscharntke [32] on conservation biological control and enemy diversity at landscape scale. Together, these highly cited works indicate that the field’s dominant theoretical and practical framing increasingly extends beyond single-enemy or single-crop interventions toward the design of agricultural landscapes that sustain natural-enemy function and deliver pest suppression as an ecosystem service.

Highly cited quantitative syntheses further reinforce this landscape and habitat-management emphasis. For example, Matthew H, Chaplin-Kramer [50] and related meta-analytic contributions synthesize evidence on crop pest and natural-enemy responses to landscape complexity, supporting the conclusion that natural-enemy effectiveness is strongly contingent on surrounding habitat composition and configuration (Table 3). More recent synthesis-driven works, including Matthias Albrecht [52] on flower strips and hedgerows, illustrate how the knowledge base has translated into specific habitat interventions that are evaluated quantitatively across systems. The prominence of these syntheses indicates that the research community places high value on evidence aggregation and generalizable inference across crops, pests, and regions.

In parallel with this landscape-centric foundation, the cited-reference profile also includes influential system-defining applied studies that anchor the field to high-impact pest problems. Notably, Nicolas Desneux [57] on the invasion and management prospects of *T. absoluta* ranks among the most cited works, indicating that crop–pest case studies with broad geographic relevance can become major reference points alongside general ecological frameworks. Additional highly cited contributions address variability and boundary conditions in biological control outcomes, including studies emphasizing inconsistent pest–predator responses and hypothesis-driven explanations for when natural habitats do not enhance pest control. These works collectively signal a mature field that not only develops habitat-based strategies but also interrogates the conditions under which such strategies succeed or fail.

The cited-reference structure suggests that biological control/IPM research is anchored by three inter-linked knowledge streams: (i) foundational habitat management concepts for conserving natural enemies, (ii) landscape complexity and biodiversity frameworks supported by quantitative syntheses, and (iii) high-impact applied case studies that connect ecological mechanisms to urgent pest management challenges. This combination of theory, synthesis, and application defines the current intellectual architecture of the field and provides a coherent basis for interpreting emerging fronts identified through keyword and burst analyses.

### 3.7. Analysis of Top Keywords and Keyword Clustering Analysis

**Figure 3:**
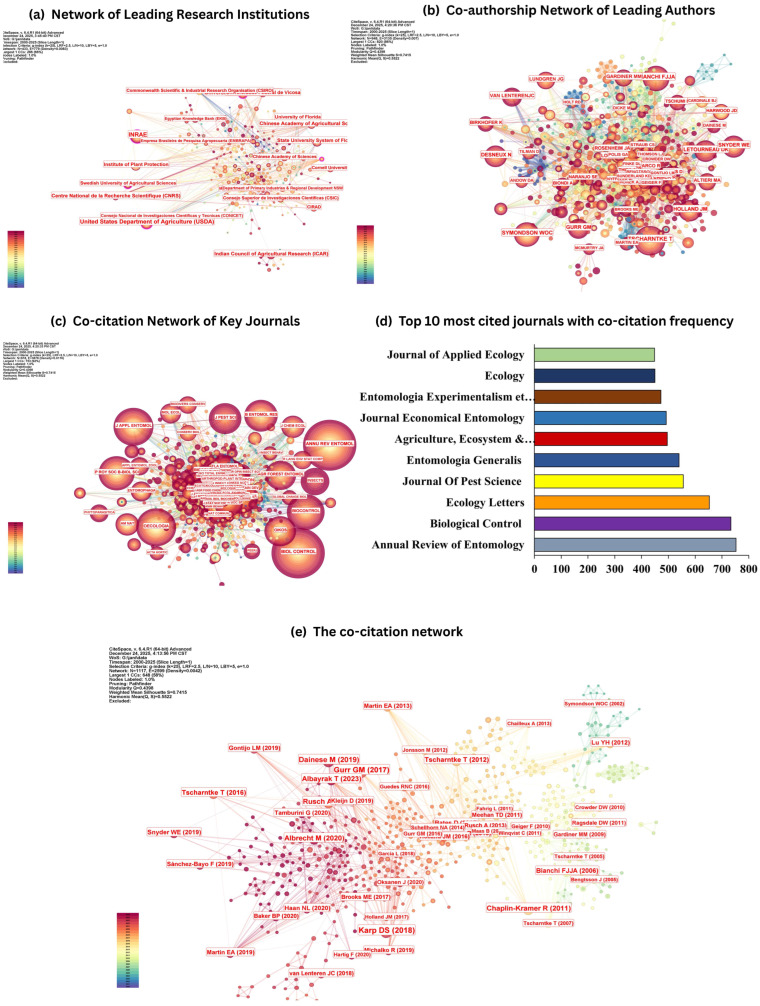
Collaboration and intellectual structure maps of biological control/IPM research (2000–2025). (**a**) Institutional collaboration network highlighting leading institutions and their linkages within the field. (**b**) Co-authorship network of leading authors, illustrating collaboration clusters and relatively distributed brokerage across groups. (**c**) Journal co-citation network showing core outlets shaping the knowledge base. (**d**) Top 10 most co-cited journals (co-citation frequency). (**e**) Cited-reference co-citation network depicting the field’s foundational references and clustered intellectual communities.

Keyword co-occurrence analysis provides a structured view of the conceptual organization and evolving emphases of biological control and integrated pest management (IPM) research across the 2000–2025 dataset (Figure 3). High-frequency keywords indicate the dominant topics that anchor the field, whereas the network structure and clustering reveal how those topics connect into coherent thematic communities. As expected, “biological control” represents the principal hub term in the network and shows the highest occurrence (approximately 560), confirming its central role as the unifying concept across the literature (Figure 3a). The prominence of “natural enemy” (approximately 300) further highlights that most studies are framed explicitly around natural-enemy-mediated pest suppression, reinforcing the long-standing positioning of predators and parasitoids as functional components of IPM (Figure 3b). Additional frequently occurring top-ten keywords such as biodiversity, management, predators, diversity, and natural enemies indicate that contemporary biological control research is increasingly embedded within ecological and agroecosystem management paradigms, rather than being restricted to single-agent or single-crop approaches.

The keyword co-occurrence map (Figure 3a) demonstrates dense connectivity among the most frequent terms, suggesting strong conceptual integration between biological control implementation and broader ecological processes. The tight coupling of “biological control” with terms related to biodiversity and management reflects an emphasis on strategies that enhance pest suppression through ecological mechanisms, including landscape configuration, habitat provisioning, and conservation-oriented interventions. Importantly, the network topology indicates that research themes are not isolated; instead, ecological terminology and applied pest control terminology are repeatedly co-mentioned, consistent with a field that increasingly frames biocontrol as an ecosystem service and a component of agroecological intensification.

The clustering analysis (Figure 3c) provides a higher-resolution interpretation of this structure by organizing keywords into distinct thematic groups. A major cluster centered on biological control and pest management implementation encompasses terms such as biological control, pest management, predators/generalist predators, and pest control, representing the applied core of the field. A second prominent cluster emphasizes agroecosystem and habitat-based management, typically capturing terms associated with agricultural landscapes, habitat management, conservation, and biodiversity-driven pest regulation. This cluster reflects the widespread focus on conservation biological control and the design of agricultural environments that support natural-enemy populations. A further theme visible in the clustering is the rise of sustainability-oriented IPM framings, including concepts aligned with ecological intensification and ecosystem services. In addition to these core clusters, peripheral clusters capture more specialized or emerging application domains, indicating diversification of biological control research into new pest systems and intervention technologies (Figure 3d).

The keyword frequency and clustering results indicate that the biological control/IPM literature is organized around a stable conceptual nucleus (“biological control” and “natural enemy”), while recent research increasingly integrates biodiversity, habitat management, and landscape-scale strategies as central components of sustainable pest suppression. The close association among these terms in the co-occurrence network suggests that ecological design and conservation-oriented approaches have become increasingly mainstream within biological control research, shaping both its dominant themes and its evolving research frontiers (Figure 3a–d).

## 4. Discussion

### 4.1. Sustainability and Ecological Pest Control: Changes in Research Trends

A limitation of the present dataset is its predominant representation of conservation biological control, with comparatively fewer documents addressing classical or augmentative biological control. This reflects the search strategy and indexing practices in Web of Science, which may underrepresent applied release-based biocontrol studies that use specific agent names or pest targets rather than the generic term “biological control.” Consequently, the following discussion interprets trends within the context of conservation biological control and habitat management, rather than claiming to represent the full breadth of biological control as a discipline.

The bibliometric patterns observed across 2000–2025 depict biological control and IPM as a field that has moved from a relatively compact early literature into a mature, rapidly expanding research domain. The sustained rise in annual output, particularly the acceleration after 2011 and the strong productivity maintained through the most recent period, signals not merely growth in volume, but an expansion in the diversity of questions, scales of inquiry, and methodological approaches used to study natural-enemy-mediated pest suppression. This trajectory is consistent with the broader shift in crop protection science toward ecologically grounded interventions and away from sole reliance on chemical control, driven by concerns over resistance evolution, non-target effects, and biodiversity losses [57,58].

A salient feature of the dataset is the clear prominence of landscape- and habitat-based framings in the intellectual base and topical structure of the literature. The dominance of highly cited foundational works on habitat management and conservation biological control indicates that ecological engineering at the field and landscape scale has become a primary conceptual lens through which biological control is understood and operationalized. The influence of Douglas A. Landis’s synthesis on conserving natural enemies through habitat management remains evident in how subsequent research has prioritized resource provisioning, refugia, and landscape complexity as determinants of enemy communities and pest suppression [48]. Similarly, Teja Tscharntke’s work on enemy diversity and landscape-scale processes reflects an enduring interest in how spatial heterogeneity shapes trophic interactions and stabilizes biological control outcomes across cropping systems [32,55]. Foundational studies on classical and augmentative biological control appeared less prominently among the most highly cited references, a pattern likely associated with the bibliometric search configuration and the indexing structure of the literature, which favored publications framed around habitat management, landscape complexity, and conservation biological control. By contrast, quantitative syntheses such as those of Chaplin-Kramer [50], Karp [53], and Rusch [54] were strongly represented, indicating sustained emphasis on broad ecological inference, context dependence, and the consistency of biological control outcomes across contrasting agroecosystems.

The keyword structure reinforces this interpretation. The centrality of “biological control” and “natural enemy” is expected, but the consistent co-occurrence of biodiversity- and management-related terms indicates that biological control is now commonly articulated as a component of ecosystem service delivery within agroecosystems rather than as a standalone tactic. This aligns with the conceptual maturation of “ecological intensification,” in which yield stability and pest suppression are pursued through functional biodiversity, habitat design, and ecological regulation rather than increasing external inputs [52]. The recent prominence of sustainability-aligned terms in the burst profile is also coherent with policy and research agendas emphasizing nature-based solutions and biodiversity-mediated benefits for crop production [52,59]. Importantly, the appearance of terms such as habitat management and conservation among emergent fronts indicates that the research frontier is not simply generating new agents but refining landscape-scale strategies that can be scaled, evaluated, and integrated into decision-making frameworks.

At the same time, the author and institutional network metrics suggest a field that is broad and productive but not strongly centralized. The low author-centrality values (0–0.01) indicate that scholarly influence and collaboration are distributed across multiple clusters rather than concentrated in a small set of “bridge” individuals. This is not necessarily a weakness; rather, it is consistent with a domain where research is strongly shaped by region-specific cropping systems, pest complexes, and policy environments, which naturally produce semi-autonomous research communities. However, such fragmentation can also slow synthesis across taxa and contexts, which may partially explain why the field continues to debate boundary conditions for effective biocontrol despite decades of research [53,55]. The institutional landscape where national research organizations and major university systems occupy prominent hub positions suggests that the capacity for large-scale, multi-region studies exists, but the translation of that capacity into tightly integrated global collaborations may remain uneven. Strong bridging institutions (e.g., USDA and INRAE in the present dataset) likely play an outsized role in enabling comparative work and cross-system synthesis, particularly when research spans temperate and tropical production settings.

The country distribution provides additional context for how biological control research is being shaped. The dominance of the United States and China in publication output is consistent with their scale of agricultural research infrastructure, but the strong presence of European contributors (e.g., France and Germany) and major agricultural economies (e.g., Brazil and India) points to broad geographic ownership of the research agenda. This matters because biological control efficacy and adoption are mediated by agroecological context, regulatory regimes, and farm structure, all of which vary substantially across regions. A globally distributed research base increases the probability that findings will be tested across climatic zones and cropping systems, but it also increases heterogeneity in study designs and endpoints. The field’s increasing reliance on meta-analysis and quantitative synthesis appears, in part, to be a response to this heterogeneity: as evidence accumulates across contexts, synthesis becomes essential for extracting decision-relevant patterns and identifying where interventions predictably succeed [50,52,54].

While parasitoids are a foundational natural-enemy guild in biological control historically central in both classical and augmentative programs, the present results, dominated by habitat/landscape and ecosystem-service framings, suggest that parasitoid-specific terminology may be less prominent at the macro-trend level than broader ecological-management constructs. This does not imply that parasitoids are unimportant; rather, it suggests that parasitoid research may frequently be indexed through system- or taxon-specific terms (e.g., target pest names, crop systems, or functional ecology descriptors) rather than the generic label “parasitoid.” It also reflects an evolution in how the field narrates biological control: contemporary work often emphasizes functional outcomes (pest suppression, stability, resilience) and enabling conditions (landscape complexity, floral resources, reduced pesticide disturbance) that apply to both predators and parasitoids [58,59,60]. This is precisely why parasitoid-focused sub-analysis is valuable: it clarifies whether parasitoid work is embedded within broader clusters or represents a distinct thematic stream, and it prevents an interpretive gap between title scope and results-driven conclusions.

The journal co-citation profile further underscores the field’s interdisciplinary structure. The prominence of review outlets such as *Annual Review of Entomology* suggests that integrative scholarship plays a major role in consolidating evidence, shaping research agendas, and translating ecological theory into management guidance. Concurrently, the centrality of specialized and applied journals such as *Biological Control*, *Journal of Pest Science*, *Agriculture, Ecosystems & Environment*, and others reflect a durable applied core oriented toward implementation, evaluation, and context-specific performance. This two-tier structure mirrors a common pattern in mission-driven disciplines: synthesis journals define frameworks and identify gaps, while applied journals accumulate system-level evidence needed for translation. Notably, the high influence of habitat- and landscape-centered articles in the cited-reference profile indicates that even the applied literature increasingly relies on ecological engineering and landscape ecology as the basis for scalable IPM strategies [31,55].

Several implications follow from these patterns. First, the field is converging on the view that biological control effectiveness is strongly contingent on landscape composition, habitat provisioning, and disturbance regimes, which together shape the abundance, diversity, and stability of natural-enemy communities [61,62,63]. Second, the prominence of ecological intensification and conservation-oriented bursts suggests increasing attention to designing farming systems that simultaneously deliver pest suppression and broader ecosystem services. Third, the influential role of quantitative syntheses indicates ongoing demand for decision rules: not simply whether “flower strips work,” but under what landscape contexts, crop systems, and management regimes they deliver net pest-control gains without unintended effects [64,65]. Finally, the distributed collaboration structure suggests that future advances will benefit from more deliberate cross-cluster integration, particularly through harmonized metrics for pest suppression, yield impacts, and enemy functional traits, so that evidence can be compared across regions and taxa with less ambiguity.

This study also has the limitations typical of bibliometric analyses. Reliance on a single database can underrepresent non-English publications, regionally indexed journals, and practitioner-oriented outputs that may be influential for implementation. In addition, keyword-based mapping is sensitive to terminology: conceptually similar work can fragment across multiple labels, and some domains (including parasitoid research) may be captured indirectly through pest- or crop-specific terms. Finally, citation-based measures favor older publications and review articles, which can amplify the apparent dominance of foundational syntheses. These limitations do not undermine the central trends reported here, but they should temper strong claims about thematic absence and motivate careful interpretation of “hotspots” as indicators of visibility rather than definitive measures of real-world adoption.

The bibliometric evidence supports a coherent narrative: biological control/IPM research has expanded rapidly over the last two decades, with an increasingly landscape- and sustainability-oriented framing that prioritizes habitat management, ecosystem services, and ecological intensification. The intellectual base is anchored by seminal habitat and landscape syntheses, while the research frontier is shaped by quantitative evaluations of habitat interventions and context dependence across agroecosystems. Strengthening parasitoid-specific mapping within this broader structure will further align the manuscript with its stated scope and improve the interpretive completeness of the results.

### 4.2. Illustrative Success Stories in Classical and Augmentative Biological Control

Despite their underrepresentation in the present bibliometric dataset, classical and augmentative biological control have produced numerous documented successes. In extensive cropping systems, parasitoids such as *Apanteles* spp. and *Cotesia* spp. have provided sustained control of lepidopteran pests including corn borers and armyworms [66,67]. In fruit and vegetable production, augmentative releases of *Trichogramma* spp egg parasitoids are widely applied for the control of lepidopteran pests [68]. Entomopathogenic fungi, including *B. bassiana* and *M. anisopliae*, have been commercialized for the control of thrips, aphids, whiteflies, and beetles in both greenhouse and field settings [69,70]. These success stories confirm that biological control encompasses a diverse toolkit beyond habitat management. Future bibliometric studies using expanded search terms (e.g., specific natural-enemy genera, “classical biological control,” “augmentative release”) would be valuable to map these complementary domains.

### 4.3. The IPM and the Role of Habitat Management

Keyword co-occurrence analysis reveals that IPM-related terms are strongly associated with “habitat management” and “ecosystem services,” indicating a shift toward landscape-level interventions within the literature. This pattern is supported by the co-citation network, where foundational works by Landis and Gurr, G.M [48,51] rank among the most highly cited references, demonstrating that habitat manipulation to conserve natural enemies remains a central concept. The prominence of these works within the intellectual base suggests that the field has converged on the view that pest suppression is enhanced when natural-enemy populations are supported through diversified vegetation, floral resources, and reduced pesticide disturbance. More recent quantitative syntheses [18,71] have reinforced this framework by providing meta-analytic evidence that habitat interventions such as flower strips and hedgerows can improve pest control outcomes when implemented under appropriate landscape and cropping conditions. Together, the bibliometric patterns indicate that habitat management has become a core component of IPM, with the literature increasingly emphasizing the design of agricultural systems that sustain natural-enemy communities as a foundation for long-term ecological resilience. Articles on manipulation of habitats are often referenced in journals such as *Annual Review of Entomology* and *Ecology Letters*, such as the ones by [48,51], One of the USDA documents issued in 2025 integrates IPM and preservation of natural resources. An ACS Omega review published in 2024 highlights the usefulness of habitat management to minimize the usage of chemical pesticides. A 2025 PMC roadmap puts emphasis on the role of habitat in enhancing biodiversity and reducing climate effects. A 2025 agronomy study on the use of practical applications such as shelterbelts describes the use of beneficial arthropod diversity in an increased manner. Such lessons make habitat management a complex instrument to IPM, which supports resilient systems and enhances the ecological balance in the long term.

### 4.4. Research Gaps and Future Directions

The clustering and burst analyses identify several areas where research activity remains limited relative to the field’s core themes. Keywords associated with climate variability, biotechnological applications, and region-specific adaptation strategies appear with lower frequency and centrality, suggesting that these topics are not yet fully integrated into mainstream biological control research. The country and institutional analyses further indicate that while major research hubs are concentrated in North America, Europe, and East Asia, representation from tropical and developing agricultural regions where pest pressures are often highest is comparatively limited. This geographic concentration may constrain the generalizability of findings across diverse agroecological contexts.

Several gaps emerge from these bibliometric patterns. First, the limited co-occurrence of climate-related terms with biological control keywords suggests that research on how climate change affects natural-enemy pest dynamics remains underrepresented, despite its relevance for long-term pest management. Second, the absence of strong keyword linkages to biotechnological tools (e.g., genetic improvement of natural enemies, precision delivery systems) indicates an opportunity to integrate emerging technologies with habitat-based approaches. Third, the relatively low centrality of developing-country institutions in the collaboration network points to a need for expanded research capacity and cross-regional partnerships to ensure that biological control strategies are tested and adapted across the full range of cropping systems where they are most needed. Addressing these gaps will require coordinated efforts to align funding priorities, harmonize experimental methods across regions and develop scalable interventions that perform reliably under variable environmental and management conditions.

## 5. Conclusions

This bibliometric review maps the evolution of conservation biological control and integrated pest management (IPM) research from 2000 to 2025, with a focus on habitat management, natural enemies, and landscape complexity. The results demonstrate that conservation biological control—emphasizing habitat manipulation and ecological intensification—has become a dominant theme in the recent literature. Publication output accelerated markedly after 2011 and remained high in the most recent period, indicating that conservation biological control has become an increasingly central research area within sustainable crop protection. Keyword co-occurrence and clustering results show that the literature is organized around a stable core (biological control and natural enemies) while increasingly emphasizing landscape- and management-oriented concepts, including habitat management, biodiversity, and ecosystem services. Citation-burst patterns and the cited-reference profile further indicate that conservation biological control, landscape complexity, and habitat manipulation remain foundational themes, supported by influential syntheses and quantitative evaluations that seek to improve the reliability and scalability of pest suppression in agroecosystems. Country and institutional analyses reveal that research output is concentrated among a small set of major contributors, led by the United States and China, with large research organizations and university systems functioning as key hubs. However, low author centrality suggests a distributed collaboration structure comprising multiple sub-communities. Future research should integrate findings from classical and augmentative biological control to provide a more complete picture of the discipline, as the present analysis is primarily representative of conservation biological control. Additionally, future work should prioritize stronger cross-region synthesis, explicit incorporation of climate variability, and integration of emerging biotechnological approaches to enhance the predictability and adoption of biological control within IPM, thereby supporting resilient and sustainable food production systems.

## Figures and Tables

**Table 1:** Top 10 authors by English literature published.

Rank	Total Published Paper	Author	Centrality
1	13	Desneux, Nicolas	0.01
2	7	Birkhofer, Klaus	0.01
3	7	Tscharntke, Teja	0
4	6	Gurr, Geoff M	0.01
5	6	Lu, Yanhui	0
6	6	Zanuncio, Jose Cola	0
7	5	Benelli, Giovanni	0
8	5	Landis, Douglas A	0
9	5	Albrecht, Matthias	0
10	5	Traugott, Michael	0.01

**Table 2:** Top 10 research institutions contributing to the field of biological control and integrated pest management (IPM).

Rank	Total Published Papers	Institutes	Centrality
1	65	INRAE	0.14
2	52	United States Department of Agriculture (USDA)	0.17
3	35	University of California System	0.11
4	33	Chinese Academy of Agricultural Sciences	0.06
5	32	Indian Council of Agricultural Research (ICAR)	0.06
6	29	Centre National de la Recherche Scientifique (CNRS)	0.08
7	28	Universidade Federal de Viçosa.	0.01
8	25	Institute of Plant Protection	0.04
9	25	State University System of Florida	0.03
10	22	University of Florida	0.02

**Table 3:** Top 10 most cited references.

Rank	Title	Author	Journal	Citation
1	“Habitat management to conserve natural enemies of arthropod pests in agriculture”	Landis [48]	*Annual Review of Entomology*	4297
2	“Biological invasion of European tomato crops by *Tuta absoluta*: ecology, geographic expansion and prospects for biological control”	N. Desneux [49]	*Journal of Pest Science*	1574
3	“Conservation biological control and enemy diversity on a landscape scale”	Tscharntke [32]	*Biological Control*	1018
4	“A meta-analysis of crop pest and natural enemy response to landscape complexity”	Chaplin [50]	*Ecology Letters*	1302
5	“Habitat management to suppress pest populations: progress and prospects”	G.M Gurr [51].	*Annual Review of Entomology*	808
6	“The effectiveness of flower strips and hedgerows on pest control, pollination services and crop yield: a quantitative synthesis”	Albrecht [52]	*Ecology Letters*	757
7	“Crop pests and predators exhibit inconsistent responses to surrounding landscape composition”	Karp D. [53]	*PNAS*	738
8	“Agricultural landscape simplification reduces natural pest control: A quantitative synthesis”	Rusch A.R [54]	*Agr. Ecosyst. Environ.*	707
9	“When natural habitat fails to enhance biological pest control–Five hypotheses”	Tscharntke [55]	*Biological Control*	694
10	“Maximizing ecosystem services from conservation biological control: the role of habitat management”	Fiedler [56]	*Biological Control*	614

## Data Availability

No new data were created or analyzed in this study. Data sharing is not applicable to this article.

## Abbreviations

The following abbreviations are used in this manuscript:

IPM	Integrated Pest Management
WoS	Web of Science
WoSCC	Web of Science Core Collection
TS	Topic Search (WoS Search Field Tag Used In The Query)
PGPR	Plant Growth-Promoting Rhizobacteria
LLR	Log-Likelihood Ratio (Used For Naming Keyword Clusters)
JCR	Journal Citation Reports
APC	Article Processing Charge
R	R statistical software
